# Acclimatory responses of the *Daphnia pulex *proteome to environmental changes. I. Chronic exposure to hypoxia affects the oxygen transport system and carbohydrate metabolism

**DOI:** 10.1186/1472-6793-9-7

**Published:** 2009-04-21

**Authors:** Bettina Zeis, Tobias Lamkemeyer, Rüdiger J Paul, Frank Nunes, Susanne Schwerin, Marita Koch, Wolfgang Schütz, Johannes Madlung, Claudia Fladerer, Ralph Pirow

**Affiliations:** 1 Institute of Zoophysiology, University of Münster, Münster, Germany; 2 Proteom Centrum Tübingen, Interfaculty Institute for Cell Biology, University of Tübingen, Tübingen, Germany

## Abstract

**Background:**

Freshwater planktonic crustaceans of the genus *Daphnia *show a remarkable plasticity to cope with environmental changes in oxygen concentration and temperature. One of the key proteins of adaptive gene control in *Daphnia pulex *under hypoxia is hemoglobin (Hb), which increases in hemolymph concentration by an order of magnitude and shows an enhanced oxygen affinity due to changes in subunit composition. To explore the full spectrum of adaptive protein expression in response to low-oxygen conditions, two-dimensional gel electrophoresis and mass spectrometry were used to analyze the proteome composition of animals acclimated to normoxia (oxygen partial pressure [*P*o_2_]: 20 kPa) and hypoxia (*P*o_2_: 3 kPa), respectively.

**Results:**

The comparative proteome analysis showed an up-regulation of more than 50 protein spots under hypoxia. Identification of a major share of these spots revealed acclimatory changes for Hb, glycolytic enzymes (enolase), and enzymes involved in the degradation of storage and structural carbohydrates (e.g. cellubiohydrolase). Proteolytic enzymes remained constitutively expressed on a high level.

**Conclusion:**

Acclimatory adjustments of the *D. pulex *proteome to hypoxia included a strong induction of Hb and carbohydrate-degrading enzymes. The scenario of adaptive protein expression under environmental hypoxia can be interpreted as a process to improve oxygen transport and carbohydrate provision for the maintenance of ATP production, even during short episodes of tissue hypoxia requiring support from anaerobic metabolism.

## Background

The planktonic crustacean *Daphnia *spp. is an important model organism for ecology, ecotoxicology and evolutionary genomics. This genus plays a central role in the planktonic food webs of standing freshwaters. These habitats exhibit pronounced variations in ambient variables such as oxygen content and temperature, both on a temporal and spatial scale. There are more or less distinctive diurnal and seasonal changes in these abiotic factors. In addition, vertical migrations expose daphnids to a wide range of different oxygen concentrations and temperatures as well. The physiology and metabolism of poikilothermic animals are strongly affected by both environmental factors [1]. Plastic adaptive responses to environmental changes include the differential regulation of gene expression, which provides specific sets of proteins for acclimation/acclimatization and, in consequence, for the maintenance of cellular function under the new ambient conditions.

A key protein of this adaptive gene control in *Daphnia *under varying oxygen and temperature conditions is hemoglobin (Hb) [2-6]. Under hypoxia or at warm temperatures, new Hb macromolecules of altered subunit composition and with an enhanced oxygen affinity [7-11] are synthesized in the fat cells and epithelial cells of the epipodites of *D. magna *(subgenus *Ctenodaphnia*) [12]. Depending on oxygen or temperature condition, seven Hb subunits are differentially expressed, which represents a remarkable example of phenotypic plasticity and functional isoform multiplicity [13]. The release of higher quantities of these newly synthesized Hb aggregates [14,15] into the hemolymph strongly improves oxygen transport from the ambient medium to the cells and restores cellular oxygen homeostasis after environmental change [16-18]

A hypoxic induction of Hb with the consequence of an improved hemolymph oxygen transport capacity under oxygen-poor conditions has also been shown for *D. pulex *(subgenus *Daphnia *sensu stricto) [19,20]. As both species, *D. magna *and *D. pulex*, inhabit similar habitats (smaller water bodies such as ponds and ditches) and show a high tolerance to hypoxic conditions, a plastic adaptive response of similar complexity as in *D. magna *may be supposed for *D. pulex *as well. So far, sequence information was only available for one globin gene in *D. pulex*[21], although biochemical studies indicate the presence of multiple subunit isoforms [22-24]. Moreover, the full spectrum of adaptive gene control under hypoxia beyond Hb expression has remained unexplored in both species, *D. pulex *and *D. magna*. The recent release of the *Daphnia pulex *genome sequence [25,26] offers the opportunity to identify these target genes. The present study aims to analyze the protein expression patterns of animals which are acclimated to normal and low ambient oxygen conditions, respectively. Two-dimensional gel electrophoresis and mass spectrometry are employed to identify a subset of the proteome induced by hypoxia with subsequent assignment of their functional role using bioinformatic tools.

## Results

**Figure 1:**
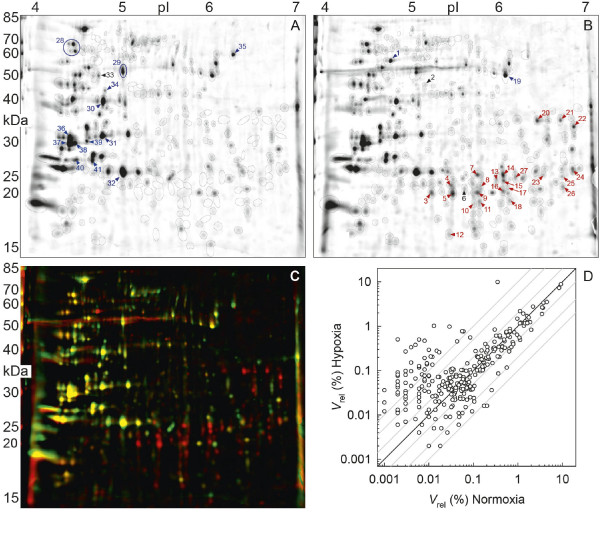
**Two-dimensional protein gels from normoxia (A) and hypoxia (B) acclimated *Daphnia pulex***. Gel images represent fusion (average) images from a set of three (A) or two (B) biological replicates. Consensus spots used for comparison are encircled. Numbers indicate spots that were picked from the 2D gels for analysis by mass spectrometry. Spots identified as globin or non-globin material were labeled in red or blue. Black labels (spots 2, 6 and 33) indicate proteins that could not be identified. (C) Dual-channel representation of the gel images shown in (A) and (B). Protein spots of similar expression intensity appear in yellow. Red indicates that spots are much stronger or unique on the gel from hypoxia-acclimated animals, whereas green means that spots are much stronger or unique in the gel from normoxia-acclimated *D. pulex*. (D) Scatter plot showing the comparison of expression levels in the two fusion images (*V*_rel_: relative spot volume). Protein spots that are strongly induced by hypoxia (approximately 50) are found in the upper left part of the graph.

Two-dimensional gels were prepared from total soluble proteins extracted from normoxic or hypoxic cultures of *Daphnia pulex *(oxygen partial pressure, *P*o_2_: 20 kPa or 3 kPa, respectively). The high reproducibility of 2D gels from the same acclimation group allowed one to generate representative fusion images for each acclimation condition (Figure 1A, B). A total of 276 spots were detected on the two fusion gels (encircled spots). The dual-channel representation of both fusion gels (Figure 1C) revealed a distinct set of up-regulated protein spots in the hypoxia-acclimation group (red-colored spots; molecular-weight range: 15–40 kDa, pI range: 5–7). In contrast, down-regulated protein spots were less obvious in the hypoxia-acclimation group as indicated by the sparse occurrence of green-colored spots in the dual-channel representation (Figure 1C). Plotting the relative volumes of related spots from both acclimation groups against each other revealed a number of approximately 50 candidate proteins that were up-regulated in the hypoxia-acclimation group (Figure 1D).

A total number of 41 spots (labeled in Figure 1A, B) comprising differentially as well as constitutively expressed proteins were excised from representative 2D gels, subjected to in-gel tryptic digestion, and analyzed by tandem mass spectrometry (MS/MS). The MS/MS data were searched against the *Daphnia pulex *protein database ("Frozen Gene Catalog" as of 03/07/2007, [26]) using the MOWSE algorithm as implemented in the MS search engine Mascot (Matrix Science Ltd. London, UK)[27]. Only in three cases (spots 2, 6 and 33 in Figure 1A, B), the identification was ambiguous. Information on identified proteins is given in the Tables 1, 2, 3 together with the hypoxia-to-normoxia expression ratio, the number and sequence of matched peptides, the percentage sequence coverage, the Mascot score (a statistical measure of identification probability), and the theoretical and experimental molecular weight (*M*_r_) and isolectric point (pI) (excluding the contribution of the signal peptide in case of extracellular proteins).

**Table 1:** Identified hemoglobins and non-identified proteins from hypoxia-acclimated (Hyp) and normoxia-acclimated (Norm) *Daphnia pulex*

**Spot no.**	**Specificity Hyp: Norm**	**Matched peptide sequences^a)^**	**Sequence coverage^b)^**	**Mascot score^c)^**	***M*_r _predicted/*M*_r _gel^d)^**	**pI predicted/pI gel^e)^**	**SP Length**	**Function^f)^** **Model name [Protein ID, Reference ID]**
20	19	FVTAHPEYQK SGLAALVAGISK KSEDLADPQTK SEDLADPQTK **LSPHMIGDVQR** NAMVSDIFIK LFKETPR QVALVADR VDTIISALDDK LLVQSLAAK GVSSDDLDSWK	30.7%	635	35.3/34	5.80/6.38	17	Hemoglobin (**Hb4**) SNAP_00002894 [234836, 42066]

21	9.3	FVTAHPEYQK SGLAALVAGISK NAMVSDIFIK LFKETPR QVALVADR VDTIISALDDK **GAWDDFGR** LLVQSLAAK GVSSDDLDSWK	26.2%	397	35.5/34	6.01/6.65	17	Hemoglobin (**Hb5**) SNAP_00002895 [234837, 311665]

22	11.3*	FVTAHPEYQK SGLAALVAGISK **KSEDLVDPQTK** **SEDLVDPQTK** LSGHMIGDVQR LFKETPR QVALVADR **LDTMIAAMDDK** **LLLDVLNAK**	24.2%	397	35.7/32	6.26/6.81	16	Hemoglobin (**Hb3**) NCBI_GNO_0400436 [311662, 311662]

3–5 7–18 23–27								proteolytic fragments of Hb

2, 6, 33								not identified

Identification was based on 2D gel electrophoresis and nano-HPLC-ESI-MS/MS analysis of trypsin-digested proteins matched against the "Frozen Gene Catalog" of the *D. pulex *protein database [26], which contains all manual curations as of July 3, 2007 as well as automatically annotated models chosen from the "Filtered Models" v1.1 reference set. The compiled information includes the spot number (Figure 1A, B), the hypoxia-to-normoxia expression ratio, the number and sequences of matched peptides, the sequence coverage, the Mascot score as a statistical measure of identification probability, the theoretical and experimental molecular weight (*M*_r_) and isolectric point (pI) of the mature protein (without signal peptide), the predicted length of the N-terminal signal peptide (SP) in extracellular proteins, the putative function of the protein, as well as the gene model name and protein identification number for the locus. The protein IDs may differ from those contained in the "Filtered Models v1.1" reference set. The Reference ID can be used to retrieve the corresponding models from this reference set. Underlined and bold-printed sequences indicate peptides that are specific for a globin gene.

a) Matched peptide sequences: tryptic peptide sequences identified *via *nano-HPLC-ESI-MS/MS.

b) Sequence coverage %: percentage of predicted protein sequence covered by matched peptides.

c)	Probability based Mascot score: -10*Log(*P*), where *P *is the probability that the observed match is a random event. Scores > 38 indicate identity or extensive homology (*p *< 0.05). Protein scores are derived from ions scores as a non-probabilistic basis for ranking protein hits. The Mascot-score calculation was performed using whole-protein sequence (including the N-terminal signal peptide in case of extracellular proteins).

d) *M*_r _predicted/*M*_r _gel: molecular mass of predicted protein/of protein on gel.

e) pI predicted/pI gel: isoelectric point of predicted proteins/of proteins on gel.

f) Function of identified proteins was obtained either *via *automated blastp search provided by JGI or after manual curation of a gene model.

* p < 0.05 (*t*-Test)

**Table 2:** Identified proteolytic enzymes from hypoxia-acclimated (Hyp) and normoxia-acclimated (Norm) *Daphnia pulex*

**Spot no.**	**Specificity** **Hyp: Norm**	**Matched peptide** **sequences**^a)^	**Sequence** **coverage**^b)^	**Mascot** **score**^c)^	***M*_r _predicted/** ***M*_r _gel^d)^**	**pI predicted/** **pI gel**^e)^	**SP** **Length**	**Function**^f)^ **Model name [Protein ID, Reference ID]**
28	1.2	DDLTETLK EPNNPDDAIPVNTAR ITTPAEDR ITTPAEDRR VIVTETDYLK VIVTETDYLKK KLVALLDATPTR LVALLDATPTR TIANYIHWR TETTLMIANLK TLVDDATWMDDGTK KDFLTLR LEEQILTDPHSPSR VIGPLSNNEDFAR	18.0%	876	75.4/72	4.36/4.4		Peptidase M13 estExt_Genewise1Plus.C_750105 [200882, 200882]
								
		IYGSYQACR YVELSNK ELHAYIR VLGVAPPVGR VWLEAENAK	6.6%	210	73.4/72	4.81/4.4	19	Peptidase M2 PASA_GEN_6000071 [307230, 307230]
								
		SGQAVEYLPGR TYTVAADDAR	3.6%	116	64.1/67	4. 66/4.4	19 (?)	Carboxylesterase, type B PASA_GEN_25200006 [304160, 304160]
								
		NADEAVAEGCNNR IVTTDIADQSK	4.6%	135	58.4/67	4.34/4.4		Sphingomyelin phosphodiesterase PASA_GEN_2900053 [304453, 304453]
								
		TYTVDGPR IVTTDIADQSK	3.6%	88	59.4/67	4.57/4.4		Sphingomyelin phosphodiesterase PASA_GEN_13800028 [301526, 301526]

31	0.8*	TFENRDMPLVK KAIVVDGGIHAR AIVVDGGIHAR NRKPNAGIGGIPCIGTDMNR KPNAGIGGIPCIGTDMNR GGAGIPFSYTVEMR DEGTFGFQLPAR QILPNNEEVWEGVK VMAESLF	22.4%	475	44.8/30	4.82/4.75	16	Carboxypeptidase A estExt_Genewise1Plus.C_150058 [195011, 195011]
								
		KAIVVDGGIHAR AIVVDGGIHAR LTAVYGTR GGAGIPFSYTVEMR VMAESLF	10.3%	246	44.8/30	4.87/4.75	16	Carboxypeptidase A NCBI_GNO_1500041 [315693, 315693]
								
		GVTDLTIFR VVAGEHSLR	6.5%	135	29.1/30	4.88/4.75	15	Trypsin SNAP_00016212 [231152, 248154]

32	0.9	IVGGTQASPNEFPYQISLR LGSHICGASIYK HEHVSYSSR GSYGTNAITDSMICAGFR	22.7%	177	26.7/23	5.43/4.98	17	Trypsin estExt_fgenesh1_kg.C_230008 [230885, 230885]

36	0.85	VVAGEHSLR SVDVPVVDDDTCNR	8.9%	149	27.2/30	4.32/4.39		Trypsin e_gw1.85.43.1 [59836, 59836]
								
		LTAAEEPTRVEIR IRNDVALIK	7.5%	80	31.5/30	5.48/4.39	15	Trypsin PASA_GEN_2900126 [304512, 304512]

37	0.85	GVTDLTIFR VVAGEHSLR VVAGEHSLRTDSGLEQNR	9.8%	159	29.1/29	4.88/4.39	15	Trypsin SNAP_00016212 [231152, 248154]
								
		VVAGEHSLR SVDVPVVDDDTCNR	8.9%	149	27.2/29	4.32/4.39		Trypsin e_gw1.85.43.1 [59836, 59836]

38	1.18	GLADADIAVFK LIWMGQYNR YYRDELAGK	10.7%	123	29.8/29	4.5/4.46	19	Placental protein 11 PASA_GEN_12200001 [301221, 301221]
								
		GLADADIAVFK LIWMGQYNR YYRDELAGK	8.0%	123	38.7/29	4.57/4.46	20	Placental protein 11 PASA_GEN_6000032 [307196, 307196]
								
		VVAGEHSLR SVDVPVVDDDTCNR	8.9%	149	27.2/29	4.32/4.46		Trypsin e_gw1.85.43.1 [59836, 59836]
								
		GVTDLTIFR VVAGEHSLR	6.5%	80	29.1/29	4.88/4.46	15	Trypsin SNAP_00016212 [231152, 248154]

39	0.92	VVAGEHSLR SVDVPVVDDDTCNR	8.9%	149	27.2/29	4.32/4.59		Trypsin e_gw1.85.43.1 [59836, 59836]
								
		GVTDLTIFR VVAGEHSLR	6.5%	120	29.1/29	4.88/4.59	15	Trypsin SNAP_00016212 [231152, 248154]

40	0.57	TTEEYYVSVQK TGGGCYSYIGR	6.5%	112	26.9/25	5.32/4.47		Astacin-like metalloprotease (ACN) FRA_fgenesh1_kg.C_scaffold_182000002 [347623, 93694]
								
		GVTDLTIFR VVAGEHSLR	6.5%	109	30.7/25	4.82/4.47	15	Trypsin SNAP_00016212 [231152, 248154]

41	1.16	LTAAEEPTR LTAAEEPTRVEVR IINDVALIK	9.1%	141	25.3/25	4.52/4.65		Trypsin e_gw1.29.198.1 [52244, 52244]

Identification was based on 2D gel electrophoresis and nano-HPLC-ESI-MS/MS analysis of trypsin-digested proteins matched against the "Frozen Gene Catalog" of the *D. pulex *protein database [26], which contains all manual curations as of July 3, 2007 as well as automatically annotated models chosen from the "Filtered Models" v1.1 reference set. The compiled information includes the spot number (Figure 1A, B), the hypoxia-to-normoxia expression ratio, the number and sequences of matched peptides, the sequence coverage, the Mascot score as a statistical measure of identification probability, the theoretical and experimental molecular weight (*M*_r_) and isolectric point (pI) of the mature protein (without signal peptide), the predicted length of the N-terminal signal peptide (SP) in extracellular proteins, the putative function of the protein, as well as the gene model name and protein identification number for the locus. The protein IDs may differ from those contained in the "Filtered Models v1.1" reference set. The Reference ID can be used to retrieve the corresponding models from this reference set. Underlined and bold-printed sequences indicate peptides that are specific for a globin gene.

a) Matched peptide sequences: tryptic peptide sequences identified *via *nano-HPLC-ESI-MS/MS.

b) Sequence coverage %: percentage of predicted protein sequence covered by matched peptides.

c)	Probability based Mascot score: -10*Log(*P*), where *P *is the probability that the observed match is a random event. Scores > 38 indicate identity or extensive homology (*p *< 0.05). Protein scores are derived from ions scores as a non-probabilistic basis for ranking protein hits. The Mascot-score calculation was performed using whole-protein sequence (including the N-terminal signal peptide in case of extracellular proteins).

d) *M*_r _predicted/*M*_r _gel: molecular mass of predicted protein/of protein on gel.

e) pI predicted/pI gel: isoelectric point of predicted proteins/of proteins on gel.

f) Function of identified proteins was obtained either *via *automated blastp search provided by JGI or after manual curation of a gene model.

* p < 0.05 (*t*-Test)

**Table 3:** Identified carbohydrate-modifying enzymes from hypoxia-acclimated (Hyp) and normoxia-acclimated (Norm) *Daphnia pulex*

**Spot no.**	**Specificity** **Hyp: Norm**	**Matched peptide** **sequences**^a)^	**Sequence** **coverage**^b)^	**Mascot** **score**^c)^	***M*_r _predicted/*M*_r _gel^d)^**	**pI predicted/** **pI gel**^e)^	**SP** **Length**	**Function**^f)^ **Model name [Protein ID, Reference ID]**
1	7.2	MFQLLNR WINGLANSK DGCDFASYR MNDHTFYGPGSTFK FYVQNGVR GLFGDLDDHK GLFGDLDDHKNK	13.3%	284	48.2/58	4.73/4.72	19	Cellubiohydrolase (**CEL7A**) PIR_PASA_GEN_1000209 [347598, 300366]

19	1.4	GNPTVEVDLTTEK MGTETYHHLKK NGKYDLDFK NPASDPATYLESNK RIQMAVDCK ACNCLLLK VNQIGTVTESIAAHK LAKYNQILR IEEELGAAAK	22.6%	468	46.8/51	5.98/6.01		Enolase (**ENO**) PIR_PASA_GEN_1500033 [347595, 301844]

29	1.2	KSILFYEAQR SILFYEAQR NAYTAAGELDNGLAALR QLYDFAK MAGISVLLSR ILGDQKYK QQIDYALGSTGR SYVVGFGNNPPVK	17.7%	355	47.3/53	5.09/5.00	18	Endo-β-1,4-Glucanase (**CEL9A**) PIR_estExt_fgenesh1_kg.C_70001 [347602, 230437]
								
		VQLEEEAEAR LTHELDKTR KLGDENAELK LKTEIQR	4.1%	124	103.7/53	5.42/5.00		Myosin estExt_Genewise1.C_2380001 [219409, 219409]

30	0.8	DSILHIKPTLTEDR GGGNTINPAMAAR YGRVEVNAK SSTPGYNSAFHR YQLEWTPDYLK FSIDDVETGR	19.8%	327	38.5/39	4.76/4.77	19	β-1,3-Glucan-binding protein (gram-negative bacteria-binding protein) PASA_GEN_0200102 [303036, 303036]
								
		SFLDFAQSK FVNWQADGVK NYYTDSCLVAAGGK	9.1%	88	39.0	4.75	19	Endo-β-1,4-Mannanase (**MAN5A**) PASA_GEN_8600009 [347627, 308762]

34	0.3*	YLGHEVGDAR LKDYYLR DLINDCIMDPK	7.3%	160	43.1/44	4.75/4.76	19	Exo-β-1,3-Glucanase (**EXG5**) PIR_PASA_GEN_1000289 [347606,300436]

35	0.5	WDDIAAECER YQPVSYK SGDENAFKSMVDR GKILEFLNK ILEFLNK LTSYGVAGFR HMWPGDLK KLSDVFHK LSDVFHK LSDVFHKK GHGGGGDLLTFR QIYNMAK	17.2%	536	54.9/62	6.03/6.30	19	α-Amylase (**AMY**) PASA_GEN_2100059 [303445, 303445]

Identification was based on 2D gel electrophoresis and nano-HPLC-ESI-MS/MS analysis of trypsin-digested proteins matched against the "Frozen Gene Catalog" of the *D. pulex *protein database [26], which contains all manual curations as of July 3, 2007 as well as automatically annotated models chosen from the "Filtered Models" v1.1 reference set. The compiled information includes the spot number (Figure 1A, B), the hypoxia-to-normoxia expression ratio, the number and sequences of matched peptides, the sequence coverage, the Mascot score as a statistical measure of identification probability, the theoretical and experimental molecular weight (*M*_r_) and isolectric point (pI) of the mature protein (without signal peptide), the predicted length of the N-terminal signal peptide (SP) in extracellular proteins, the putative function of the protein, as well as the gene model name and protein identification number for the locus. The protein IDs may differ from those contained in the "Filtered Models v1.1" reference set. The Reference ID can be used to retrieve the corresponding models from this reference set. Underlined and bold-printed sequences indicate peptides that are specific for a globin gene.

a) Matched peptide sequences: tryptic peptide sequences identified *via *nano-HPLC-ESI-MS/MS.

b) Sequence coverage %: percentage of predicted protein sequence covered by matched peptides.

c)	Probability based Mascot score: -10*Log(*P*), where *P *is the probability that the observed match is a random event. Scores > 38 indicate identity or extensive homology (*p *< 0.05). Protein scores are derived from ions scores as a non-probabilistic basis for ranking protein hits. The Mascot-score calculation was performed using whole-protein sequence (including the N-terminal signal peptide in case of extracellular proteins).

d) *M*_r _predicted/*M*_r _gel: molecular mass of predicted protein/of protein on gel.

e) pI predicted/pI gel: isoelectric point of predicted proteins/of proteins on gel.

f) Function of identified proteins was obtained either *via *automated blastp search provided by JGI or after manual curation of a gene model.

* p < 0.05 (*t*-Test)

In some cases, MS data suggest that more than one protein was present in the excised spot. For example, spot 29 corresponding to an apparent *M*_r _of 53 kDa yielded two proteins, an endo-β-1,4 glucanase (predicted *M*_r_: 47.3 kDa; identification based on 8 peptides) and myosin (predicted *M*_r_: 103.7 kDa; 4 peptides). Accordingly, spot 29 contains the glucanase as the major protein with a minor amount of a myosin fragment. The deviation between predicted and experimental *M*_r_/pI suggests that the fragment resulted from proteolytic cleavage during sample preparation.

The identified proteins can be classified into three groups with (i) a set of hemoglobin (Hb) subunits and fragments which were up-regulated at hypoxia acclimation, (ii) a set of proteases which were expressed in high amounts at both acclimation (oxygen) conditions, and (iii) a set of carbohydrate-modifying enzymes, for which a complex regulation pattern was observed including constant expressions as well as up- and down-regulations.

### Hemoglobins

**Figure 2:**
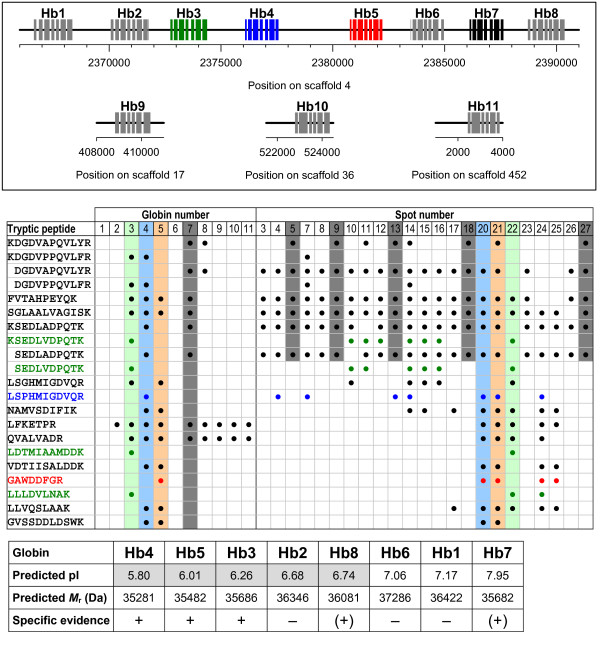
**Assignment of protein spots to the globin genes (HbA-HbL) of *D. pulex***. Positional information on the globin genes (Hb1–Hb11) is given on top (boxes represent exons). Genes with specific proteomic support (Hb3, Hb4, Hb5) are shown in green, blue and red colors. The middle part lists the tryptic peptides in the order of their appearance in the globin sequences. Black circles indicate the occurrence of tryptic peptides in the globin sequences and in the analyzed spots. Colored sequences and circles indicate tryptic peptides that are specific for only one globin. Shaded in gray is a set of six tryptic fragments which were detected in several spots (5, 9, 13, 18, 27) and which could all be assigned to subunit Hb7. The lower part lists the characteristics of globins in terms of predicted isoelectric point (pI) and molecular mass (*M*_r_). Shaded in gray are the predicted pI values which fall within the pH 4–7 gradient range used for isoelectric focussing.

Among the proteins up-regulated in hypoxia-acclimated animals, 23 spots were identified to contain Hb (Figure 1B, spots 3–5, 7–18 and 20–27). The tryptic peptides (fragments) used for the identification of Hb are listed in Figure 2 in the order of their appearance in the globin genes. Peptide sequences that are specific for one globin gene, and which therefore allow for a discrimination between globin subunits, are printed in green, blue and red colors. Only the subunits Hb3, Hb4 and Hb5 received specific support by the MS analysis of fragments. An unambiguous discrimination was not possible for the subunits Hb7 and Hb8, which received the support by the same pair of tryptic peptides. However, the spots 5, 9, 13, 18 and 27 yielded the same set of six tryptic fragments (including the Hb7/Hb8-related pair) which could all be assigned to subunit Hb7. Subunit Hb8, in contrast, was only supported by the Hb7/Hb8-related pair. This suggests that subunit Hb7 rather than Hb8 is expressed under hypoxic conditions. The spots 20, 21 and 22 mainly contained tryptic peptides related to subunit Hb4 (11 of 13 fragments), Hb5 (9 of 14 fragments) or Hb3 (9 of 11 fragments), respectively, with a sequence coverage of up to 30% (Figure 2, Table 1). In addition, the experimental *M*_r _of only these three spots (20, 21, 22) matched the expected size of an intact globin subunit (Table 1). Taking further into account the correlations between the observed and predicted pI patterns, then an assignment of subunits Hb4, Hb5, and Hb3 to the spots 20, 21 and 22 seems plausible (see Discussion). These subunits showed a 9-19-fold increase in expression under hypoxia.

### Proteases and Peptidases

Proteolytic enzymes were identified in spots 28, 31, 32, and 36–41 (Table 2). All of these nine spots were expressed in comparable amounts under both oxygen conditions. The trypsin-containing spots (31, 32, 36–41) were expressed in large amounts (Figure 1A). Several other proteases were also present including the peptidases M13 and M2 (spot 28), a carboxypeptidase A (spot 31), and an astacin-like metalloprotease (spot 40).

### Glycoside hydrolases

Several enzymes involved in carbohydrate metabolism were identified in the gels (Figure 1A, B and Table 3). A cellubiohydrolase (CEL7A, spot 1) showed the strongest differential expression with a seven-fold up-regulation under hypoxia. Acclimation to hypoxia was additionally associated with a slight up-regulation of the enolase (ENO, spot 19). The strongest reduction in protein expression was observed for an α-amylase (spot 35) and an exo-β-1,3-glucanase (EXG5, spot 34).

## Discussion

Using a proteomic approach, the present study identified, aside from constitutively expressed proteins, a set of proteins, which is differentially expressed in normoxia or hypoxia acclimated *Daphnia pulex*. Identification and biochemical characterization of this proteome subset may contribute to the ongoing annotation process of the *D. pulex *genome as it provides complementary information on the protein level for predicted genes with EST support as well as information on key players of adaptive gene control involved in the adjustment of physiological functions under different oxygen conditions.

### Methodical constraints

To improve resolution, proteins were separated on 2D gels using a relatively narrow pH gradient (pH 4–7). Although the pI of the bulk of soluble proteins falls into this pH region during isoelectric focusing, there is still a considerable number of polypeptides with pI values outside this range (unpublished data), which requires additional experiments in future with extended pH gradients for isoelectric focusing. In order to guarantee identical protein load per gel, a relatively low amount of protein was loaded (140 μg/gel). Therefore, protein identification by mass spectrometry was possible only for prominent spots representing high-copy proteins. The identification of differentially expressed proteins was impeded by a residual proteolytic activity, since several spots were identified as cleavage fragments of proteins (see below), despite of the use of protease inhibitors for protein extraction and the preparation at cold temperatures. The residual proteolytic activity in the crude extract is the consequence of the high abundance of proteases, which are equally expressed under both normoxic and hypoxic conditions. Hence, for future experiments a custom made inhibitor cocktail for specific and effective inhibition of *D. pulex *proteases has to be developed.

### Oxygen transport and energy metabolism

The analysis of differential expression patterns revealed the hemoglobins as one major group that is induced under hypoxia. Eight genes coding for the globins AHb1–Hb8 are present in the genome of *Daphnia pulex *forming a cluster on scaffold 4, whereas three additional gene copies are spread across different scaffolds (Figure 2) [28]. Among the 23 Hb spots, sequences specific for the subunits Hb3, Hb4 and HB5 were detected by the MS analysis of fragments. The tryptic-peptide analysis further revealed that subunit Hb7 (but not Hb8) is very likely expressed under hypoxic conditions. The spots 20, 21 and 22 showed a dominance of tryptic peptides related to subunit Hb4 (11 of 13 fragments), Hb5 (9 of 14 fragments) and Hb3 (9 of 11 fragments), respectively, with a sequence coverage of up to 30% (Figure 2, Table 1). As the mature subunits have predicted pI values of 5.80–7.95 and *M*_r _values of 35–37 kDa (Figure 2), they should distribute according to their pI values along a horizontal line in the order Hb4-Hb5-Hb3-Hb2-Hb8-Hb6-Hb1-Hb7. Due to the pH 4–7 gradient range used for isoelectric focusing, however, only the subunits Hb4, Hb5, Hb3, Hb2 and Hb8 would migrate into this pH range. In addition, the experimental pI values (Table 1) turned out to be shifted by 0.5–0.7 units towards higher values in comparison to the predicted pI values (Hb4: 5.80→6.38, Hb5: 6.01→6.65, Hb3: 6.26→6.81), which maybe due to posttranslational modifications of the Hb subunits [15]. Provided that such a pI shift applies to all other products of the globin gene cluster as well, then none of the remaining subunits (Hb2, Hb8) would have migrated into and would be visible in the pH 4–7 gradient range. Actually, only the spots 20–22 showed experimental *M*_r _values (Table 1), which matched the expected size of intact globin subunits. Consequently, the spots 20, 21 and 22 most likely represent the subunits Hb4, Hb5 and Hb3. The contamination of these spots with small quantities of unrelated tryptic peptides could be the consequence of minor proteolytic cleavage of other subunits and the co-localization of cleavage products of similar pI at these spots.

Some protein spots (spots 5, 9, 13, 18, 27) within the molecular-weight range of 15–30 kDa (Figure 1B) yielded tryptic peptides that very likely represented cleavage products of subunit Hb7 (Figure 2). Further low-molecular weight spots in extracts from hypoxia-acclimated *D. pulex *contained fragments of conserved sequences that could originate from any of the subunits Hb3, Hb4, Hb5, and Hb7 (or Hb8) (Figure 2).

All three spots of intact hemoglobin subunits (spots 20–22) showed an increase in intensity in gels of hypoxia-acclimated animals. The induction intensity ranged from 9-fold (Hb5) to 19-fold (Hb4), which is comparable to the hypoxia-induced increase (5–21-fold) of Hb concentration in the hemolymph of *Daphnia magna *[9,10]. The support for the globins Hb3, Hb4, Hb5 and Hb7 suggests that these subunits are dominant in hypoxia-acclimated animals. A similar dominance of only a few subunits was found in hypoxia-acclimated *Daphnia magna *[10]. However, we cannot fully exclude the presence of other globins, because the migration position of intact chains of these subunits is outside the pH 4–7 gradient range, and the concentration of proteolytic cleavage products with pI values smaller than 7 could be too low to be detectable by protein gel staining or mass-spectrometric analysis.

The mechanism of hypoxic Hb induction involves the transcription factor HIF (hypoxia inducible factor) in *Daphnia magna *[29]. Similar as in mammalian cells [30], HIF is prevented from degradation under oxygen-poor conditions and binds to enhancing elements present in the intergenic regions of *Daphnia*'s Hb gene cluster[9,28]. The target genes in vertebrates include proteins involved in oxygen homeostasis (EPO, VEGF) as well as key players of carbohydrate metabolism (for reviews, see [31,32]). The latter enzymes are involved in anaerobic metabolism which guarantees ongoing energy provision during oxygen deprivation. Since anaerobiosis is a less effective mode of ATP production, it requires a higher turnover rate of glycosides, which can be guaranteed by an increase in the concentration of glycolytic enzymes.

It is therefore reasonable to assume that glycolytic enzymes experience a comparable induction in animals exposed to environmental hypoxia. However, the present study identified only one element of the HIF-target genes involved in glycolysis, the enolase (ENO), which was only slightly induced (factor 1.4) in hypoxia-acclimated *D. pulex*. HIF-binding sites (hypoxia responsive elements: HRE) are present upstream of the enolase gene. The motif ACGTGT can be found in cis positions at -173 and -481. At least the first one is within the functional range where HIF-binding affects gene expression, as was documented for hypoxic *D. magna *hemoglobin induction [29]. The only moderate induction of enolase might be the consequence of the increase in oxygen-transport capacity arising from the strongly elevated Hb concentration. The successful restoration of oxygen homeostasis may reduce the need for adjustments in protein expression. A stronger induction than found here might be observed in animals from acute hypoxic exposure. The adjustment of the oxygen-transport system to environmental hypoxia *via *Hb induction, however, does not exclude the possibility of an occurrence of hypoxic states within certain cells and tissues. Episodes of higher energy demand, e.g. during enhanced activities, may drive the oxygen-transport system to the limit, thereby increasing the risk of oxygen lack in specific body regions. Moreover, the oxygen supply of cells depends on their size or location. Particularly in large cells (with small surface-to-volume ratio) or cells with a high metabolic rate, the *P*o_2 _threshold for the activation of anaerobic metabolism and the stabilization of HIF may be passed more or less frequently. The fat cells, for example, which constitute one major site of Hb synthesis in *Daphnia *[12], are likely to be the first candidates which suffer from hypoxia. The risk for undersupply with oxygen arises from their large size and their distribution in the body core region, where hemolymph *P*o_2 _values are low [18]. So, the difference in the up-regulation of Hb and other HIF target genes may be related to more frequent hypoxic episodes in Hb-synthesizing tissues.

Enolase is known to be one of the most abundantly expressed cytoplasmic proteins [33]. The dimeric magnesium-containing enzyme catalyzes the conversion of 2-phosphoglycerate to phosphoenolpyruvate. Besides its role in glycolysis, it has been characterized as a stress protein involved in hypoxia and thermal tolerance; even a heat-shock protein function has been reported [33]. In *D. pulex*, the enolase is present in high amounts (spot 19). The slight induction of this enzyme under hypoxia is well in line with its regulation by HIF, its role in anaerobiosis, and its possible function as a stress protein. Its high expression already in normoxia-acclimated animals might be interpreted as a pre-adaptive feature which renders a marked hypoxia response unnecessary.

### Proteolytic enzymes

A group of proteolytic enzymes (particularly trypsin; spots 28, 31, 32, 36–41) was identified in large amounts in all 2D gels of *D. pulex*. Their expression was unaffected by hypoxia acclimation. In *D. magna*, the largest portion of proteases are trypsin- and chymotrypsin-like enzymes [34], which are endopeptidases characterized by the presence of a serine residue in the active site. More than 98% of the proteolytic activity of *D. magna *can be found in the gut. In the whole-animal extracts used in the present study, intestinal digestive enzymes are included in the preparation. *Daphnia*'s serine proteases are targets of common inhibitors [34]. Specific inhibition of serine proteases is reported to reduce the total proteolytic activity of *Daphnia *to 15%, indicating that the residual proteolytic activity may originate from non-serine proteases [34]. Our identifications included indeed other classes of digestive enzymes such as the astacin-like zinc metalloendopeptidase (spot 40) [35,36], the zinc metallopeptidase M13 (spot 28), which is probably a membrane-bound enzyme because of the absence of a signal peptide in the predicted protein sequence, and the secretory zinc metallopeptidases M2 (spot 28), which carries signatures of a dipeptidyl carboxydipeptidase [37]. Strong expression was also observed for the zinc carboxypeptidase A (spot 31), which is secreted as an inactive proenzyme that becomes activated by the cleavage of an N-terminal propeptide [37]. This activating cleavage may explain the discrepancy between the predicted *M*_r _(44.8 kDa for the mature protein with propeptide but without signal peptide) and the measured *M*_r _of 30 kDa. While the protease-inhibitor cocktail used in the present study contained specific inhibitors to block serine proteases and metalloproteases, it seems that the inhibition was incomplete and that not all types of proteases were covered by the chosen inhibitors. Moreover, the extraction of proteins at cold temperatures might not have been as effective as expected. Since daphnids are confronted to large temperature fluctuations in the natural habitat, it is possible that their proteases are adapted to operate over a wide range of temperatures. Irrespective of these methodical aspects, the high representation of proteases in the *D. pulex *proteome documents an enormous digestive capacity, which probably guarantees an optimal exploitation of food resources to support the high growth and reproduction rates which are characteristic for these animals.

### Polysaccharide-degrading enzymes

A set of polysaccharide-degrading enzymes was identified in the 2D gels. The putative enzymatic specificities, which could be assigned by sequence similarity with classified glycosyl hydrolases [38-40], comprise the hydrolytic cleavage of endoglycosidic bonds in α-1,4-glucans (α-amylase, spot 35), β-1,4-glucans (endo-glucanase, CEL9A; spot 29), and β-1,4-mannans (endo-mannanase, MAN5A; spot 30) as well as the exoglycosidic cleavage of β-1,4-glucans (cellubiohydrolase, CEL7A; spot 1) and β-1,3-glucans (exoglucanase, EXG5; spot 34). These different glycosidic bonds are characteristic of storage polysaccharides (starch: α-1,4-linked glucan) and structure polysaccharides (cellulose: β-1,4-glucans; hemicellulose: β-1,4-mannans and others) of plants including nanoplanktonic green algae, the typical food of daphnids [41]. β-1,3-glucans are structural components in the cell wall of fungi and algae. These functional assignments, the high degree of expression (Figure 1), and the presence of an N-terminal signal peptide (Table 3) strongly suggest that these candidate proteins are secretory digestive enzymes involved in the degradation of storage and structural polysaccharides.

The origin of cellulase activity in multicellular animals was formerly assigned to symbiotic microorganisms living in the host's gastrointestinal tract (see [42] for a review). The discrimination of cellulolytic enzymes from symbionts and their hosts by functional analyses is still a difficult task [43,44], but there are many indications for an endogenous (i.e. non-symbiontic) cellulolytic activity in metazoans including crustaceans [45-48]. Molecular biology techniques provided unequivocal support for the presence of cellulase genes in various metazoan lineages such as arthropods (crustaceans and insects), annelids, ascidian chordates, echinoderms and molluscs [42,49].

Experimental support for a cellulolytic activity in daphnids was first provided by [43]. Toxicological studies in *D. magna *showed an inhibition of amylase/cellulase activities by cadmium and mercury as well as an activity increase upon chromium exposure [50]. Reduced activities of both enzymes were found under ultraviolet radiation [51]. Microarray studies [52] revealed an up-regulated expression of cellulase and amylase genes under cadmium stress. In the present study, the acclimation of *D. pulex *to hypoxic conditions was associated with a strong increase in cellubiohydrolase expression (spot1) and a moderate decrease in α-amylase (spot 35) and exo-β-1,3-glucanase (spot 34) expression. The presence of six glycosyl hydrolases among the spots of major intensity shows a large capacity for carbohydrate digestion, which obviously adapts *D. pulex *to hypoxic conditions. As suggested for protein digestion, the high potential for carbohydrate degradation may reflect a high turnover of nutrients for the animals' fast growth and reproduction rates. This suggestion might be in conflict with the 20% reduction in the oxygen-consumption rate of hypoxia-acclimated *D. magna *compared to normoxia-acclimated animals [53]. However, reduction in oxygen uptake does not necessarily imply a reduced need for digestive processes. In case of anaerobic energy production, the metabolic flux rate through the glycolytic pathway has to be increased due to the lower ATP yield of anaerobic glycolysis, which leads to an enhanced demand for carbohydrates. Concerning the regulation of the whole set of carbohydrate-degrading enzymes, the complex pattern of adaptive gene control certainly needs further investigation.

## Conclusion

Adjustments of protein expression due to hypoxia acclimation in *Daphnia pulex *include a strong induction of Hb to adapt the oxygen-transport system to an oxygen-depleted environment. Other HIF target genes such as that for enolase, which is involved in anaerobic metabolism, are induced to a lower extent. This may reflect, on the one hand, the general restoration of oxygen-transport capacity by Hb induction and, on the other hand, tissue-specific variations in cellular oxygen supply with more frequent episodes of tissue hypoxia, especially in the body core region. In contrast to carbohydrate-degrading enzymes, the set of proteolytic enzymes does not respond to hypoxia. Independent of ambient oxygen conditions, the animals maintain a high level of proteolytic power, which is probably related to the high energy demands for activity, growth and reproduction. Among the complex pattern of adaptive gene control for carbohydrate hydrolysis, the enhanced need for carbohydrates during periods of anaerobiosis is probably related to the strong hypoxic induction of cellubiohydrolase, which may serve for a degradation of structural polysaccharides.

## Methods

### Acclimation conditions

Water fleas, *Daphnia pulex*, were originally obtained from a flooded eutrophic quarry at Gräfenhain (near Dresden, Germany) [54] and have been kept in the laboratory since 2002. The animals were cultured in 1.5L M4 medium [55] in 2–3L preserving jars under a 16 h:8 h L:D photoperiod as previously described [53]. The animals were acclimated at least for three weeks (mostly months) to normoxia (100% air saturation; oxygen partial pressure, *P*o_2_: 20 kPa) or hypoxia (15% air saturation; *P*o_2_: 3 kPa) at 20°C. Normoxic medium was obtained by mild aeration using an aquarium pump, whereas hypoxic conditions were established by reducing the atmospheric pressure in the residual air space of the closed preserving jar to 15% of standard atmospheric pressure using a vacuum pump (PC 511, Vacuubrand, Wertheim, Germany). Animals were fed with green algae (*Desmodesmus subspicatus*) *ad libitum *(> 1 mg C L^-1^) every second day. Three-quarter of the medium was renewed once weekly. Any males and ephippial females were sorted out to maintain parthenogenetic reproduction.

### Protein extraction

Total (soluble) proteins were extracted from shock-frozen *D. pulex *(150–200 mg fresh weight per biological replicate). Before freezing, the animals had not been fed with algae for 12 h. The biological material was mixed 1:3 (w/v) with a freshly prepared rehydration solution containing 8 M urea, 2 M thiourea, 4% (w/v) CHAPS, 65 mM DTT, 0.5% (v/v) ampholyte-containing IPG buffer pH 4–7 (GE Healthcare, Munich, Germany), and a protease-inhibitor cocktail (Complete Mini, Roche, Mannheim, Germany) (one tablet per 10 mL solution). The biological material was disrupted using a tissue grinder (Pellet Pestle; Kimble/Kontes, Vineland, NJ, USA) for 1 min on ice, and the insoluble fraction was then removed by centrifugation at 17900 × *g *for 15 min at 4°C. The supernatant containing the soluble protein fraction was then subjected to ultrafiltration (17900 × *g *for 45 min at 4°C) using centrifugal filter devices with a molecular mass cut-off of 300 kDa (Microcon YM-300, Millipore, Schwalbach, Germany). The proteins in the lower-molecular-weight filtrate were precipitated with 13% TCA, incubated on ice for 70 min, and then centrifuged at 17900 × *g *for 15 min at 4°C. The protein pellet was repeatedly washed with ice-cold 80% acetone and centrifuged (17900 × *g *for 5 min at 4°C) ten times, and then resuspended in 200 μl rehydration solution. Protein quantification was performed using the Bradford assay [56].

### Two-dimensional gel electrophoresis

Isoelectric focussing (IEF) was performed with 142 μg of protein extract diluted in 350 μl rehydration solution using 18-cm linear pH 4–7 IPG gradients (GE Healthcare) and the Ettan IPGphor II isoelectric focusing unit (Amersham Biosciences, Uppsala, Sweden). Rehydration of the IPG strips was performed at 50 V for 11 h at 20°C. The voltage settings of the IEF comprised a 50–100 V gradient for 1 min, 100 V for 2 h, 100–1000 V gradient for 10 min, 1000 V for 30 min, 1000–4000 V gradient for 1 h, 4000 V for 30 min, 4000–8000 V gradient for 45 min, 8000 V for 4.5 h, to a final setting of approximately 46000 Vh. After IEF, the strips were equilibrated for 15 min in equilibration solution (0.05 M Tris, 6 M urea, 30% glycerol, 2% SDS, pH 8.8) containing 65 mM DTT followed by 15 min in equilibration solution containing 135 mM iodoacetamide to block free thiol groups. For the second dimension, protein separation on the basis of the molecular mass was performed using 12% polyacrylamide gels (0.56 M Tris, 0.1% SDS, pH 8.8; 20 × 18 × 0.1 cm^3^) and the Protean II xi Cell apparatus (Bio-Rad Laboratories, Munich, Germany). The PageRulerTM Protein Ladder (Fermentas, Burlington, Canada) covering a molecular mass range from 10 kDa to 200 kDa was used for molecular mass calibration. Electrophoresis was performed at 15 mA per gel for 18–21 h. After electrophoresis, gels were stained with SYPRO Ruby protein gel stain (Bio-Rad) according to the manufacturer's instructions. Stained gels were scanned with a Typhoon 9400 fluorescence imager (GE Healthcare) and analyzed with Delta2D software, version 3.5 (DECODON, Greifswald, Germany) [57]. Gels were warped manually using the exact warp mode prior to spot detection and editing.

### Statistical analysis of protein expression

Protein expression was quantified by translating the normalized intensity of candidate spots in 2D gels into relative spot volumes. Statistical differences in protein expression between the two acclimation groups were assessed by *t*-tests after differences in variance had been checked by *F*-tests.

### nano-HPLC-ESI-MS/MS

Spots of sufficient size and staining intensity (relative spot volume, *V*_rel _> 0.1%) were chosen for subsequent mass-spectrometric analyses if they were identified as differentially expressed between normoxia-acclimated and hypoxia-acclimated animals. Some spots of high but constitutive expression were also excised from representative gels. They were subjected to in-gel digestion using trypsin (sequencing grade, Promega, Mannheim, Germany) overnight at 37°C. Reversed-phase nano-LC-MS/MS was performed using an Ultimate nanoflow LC system (Dionex LC Packings, Idstein, Germany) containing the components Famos (autosampler), Switchos (loading pump and switching valves), and Ultimate (separation pump and UV-detector). The LC system was coupled to a QSTAR Pulsar i hybrid QqTOF mass spectrometer (Applied Biosystems/MDSSciex, Darmstadt, Germany), equipped with a nanoelectro-spray ion source (Column Adapter [ADPC-PRO] and distal coated SilicaTips [FS360-20-10-D-20], both from New Objective, Woburn, USA). Briefly, the tryptic peptide mixtures were autosampled at a flow rate of 30 μl/min in 0.1% aqueous trifluoroacetic acid, and desalted on a PepMap C18 trapping cartridge (LC Packings). The trapped peptides were eluted and separated on the analytical column (PepMap C18, 75 μm i.d. × 15 cm; LC Packings) using a linear gradient of 7–50% solvent B (acetonitrile 84% [v/v] in 0.1% [v/v] formic acid) for 27 min at a flow rate of 220 nl/min, and ionized by an applied voltage of 2200 kV to the emitter. The mass spectrometer was operated in the data-dependent acquisition mode to automatically switch between MS and MS/MS. Survey MS spectra were acquired for 1.5 s, and the three most intense ions (doubly or triply charged) were isolated and sequentially fragmented for 1.5 s by low-energy collision-induced dissociation. All MS and MS/MS spectra were acquired with the Q2-pulsing function switched on, and optimized for enhanced transmission of ions in the MS (*m*/*z *400–1000) and MS/MS (*m*/*z *75–1300) mass ranges. All results from 2-dimensional electrophoresis and mass spectrometry as well as all search results where stored in a LIMS-database (Proteinscape 1.3, Bruker Daltonics, Bremen, Germany).

### Identification and characterization of proteins

Proteins were identified by correlating the ESI-MS/MS spectra with the "Frozen Gene Catalog" of the *D. pulex *protein database [26] using the MOWSE-algorithm as implemented in the MS search engine (Matrix Science Ltd., London, UK) [27]. The "Frozen Gene Catalog" contains all manual curations as of July 3, 2007 as well as automatically annotated models chosen from the "Filtered Models" v1.1 set. "Filtered Models" is the filtered set of models representing the best gene model for each locus. The putative function of identified proteins was inferred by sequence homology either from the automated blastp search provided by Joint Genome Institute [26] or after manual curation of gene models. Derived protein sequences were checked for the presence of N-terminal signal sequences [58,59]. The theoretical molecular weight (*M*_r_) and isolectric point (pI) of mature proteins (without N-terminal signal peptide) was calculated using the ExPASy proteomics tool "Compute pI/MW" [60-62].

## Abbreviations

EPO: erythropoetin; *M*_r_: molecular weight; pI: isolectric point; *P*o_2_: oxygen partial pressure; VGEF: vascular endothelial growth factor.

## Authors' contributions

SS and MK were involved in the culturing of animals and performed the protein extraction as well as the 2D-PAGE. 2D-gel image analysis was carried out by TL, WS, BZ and RP. JM and CF were responsible for mass spectrometry and protein identification. RP and SS retrieved the information contained in the Tables. Figures were designed by RP. The annotation of identified genes was performed by FN and RP. BZ, RJP, and RP conceived and coordinated the study, and prepared the manuscript. All authors read and approved the final manuscript.
